# Hydatid disease of pancreas: A case report

**DOI:** 10.1556/1646.11.2019.05

**Published:** 2019-04-01

**Authors:** János Deák, Gergely Zádori, Adrienn Csiszkó, László Damjanovich, Zsolt Szentkereszty

**Affiliations:** 1 Clinical Center, Institute of Surgery, University of Debrecen, Debrecen, Hungary

**Keywords:** hydatidosis, pancreas, cyst, echinococcus, surgery

## Abstract

Primary pancreatic hydatid disease is extremely rare. Diagnosis of the disease is difficult because hydatid cysts can be confused with a pseudocyst or neoplastic cystic diseases. Authors report a case of a surgically treated hydatid disease of the uncinate process of pancreas. In a 34-year-old patient with minor symptoms, a cystic disease of the pancreas was accidentally identified. CT scan revealed a multivesicular cystic mass with a maximum of 13-cm diameter and with a calcificated wall. During laparotomy, the uncinate process of pancreas was resecated and the cystic lesion was enucleated. Patient was recovered without complications and recurrence of the disease. There must be a suspicion of hydatid disease when cysts are identified in good conditioned, asymptomatic patients, or in case of wall calcification or multivesicular cysts revealed by radiological images. Surgical procedures are recommended in uncertain diagnoses too, because differentiation preoperatively between cystic pancreatic lesions is often impossible.

## Introduction

Hydatid disease is caused by the larval stage of *Echinococcus granulosus*. The most commonly affected organs by hydatid disease are the liver and the lung. Other organs, e.g., muscles, kidneys, brain, bones, pancreas, and spleen, are rarely involved [1].

Primary pancreatic hydatid cysts are very rare. Diagnosis of the disease is difficult because hydatid cysts can be confused with pseudocysts or neoplastic cystic diseases of the pancreas. The incidence of primary pancreas hydatidosis is 0.2%–2% of all hydatid disease in endemic countries [1–3]. The cysts can be found mainly in the pancreas head, followed by the corpus and the tail region [4].

Authors report a case of a surgically treated pancreatic hydatid disease, which caused minor abdominal symptoms, and the final diagnosis of the disease was made intraoperatively.

## Case Presentation

**Fig. 1.**
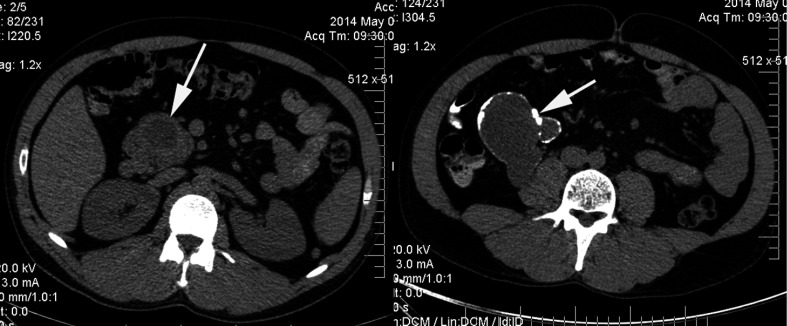
Preoperative CT scan. A cyst originated from the uncinate process of pancreas (indicated by arrow) is shown on the left slice. The right slice is taken from a lower part of the abdomen. Arrow indicates a multivesicular cyst with wall calcification

A 34-year-old male patient’s case history was examined with no any of serious diseases. The patient had complained about lower abdominal pain for a year. Ultrasonography (US) revealed a 6 × 4 × 4 cm solitary cystic lesion with clean wall in the pancreas head region. Connection between pancreas and the cyst could not have been clearly identified by US. Tumor marker (CA 19-9) levels were in the normal range. Patient applied for control only 12 months after the first examination. At that time, computed tomography (CT) scan revealed a multivesicular cystic mass with a maximum of 13-cm diameter with a calcificated wall *(Fig. 1)*. CT identified the contact between the lesion and the pancreas head. Radiological imaging did not reveal any cystic lesion neither in the liver, nor in other parenchymal organs. At this time, the patient had only abdominal distress.

During physical examination, a 10-cm diameter resilient swelling could be palpated in the epigastric region. Liver enzymes, amylase, lipase, and inflammation markers were in normal range. Blood eosinophilia could not been observed. Echinococcus serology test was negative by enzyme-linked immunosorbent assay. There was a possibility of pancreas cyst, pseudocyst, and hydatid disease as well. In regard to the size of the lesion and the uncertain diagnosis, a laparotomy was offered to the patient.

**Fig. 2.**
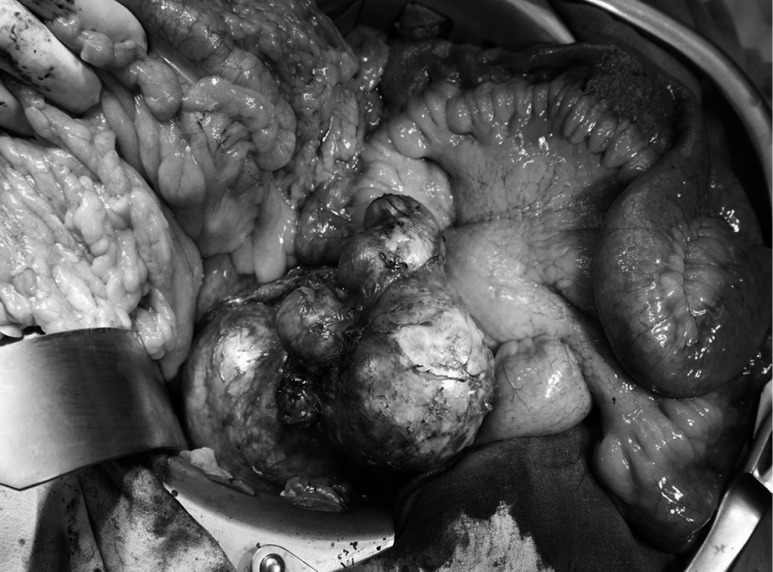
The transverse colon (left), the jejunum (right), and the multivesicular cystic lesion originated from the uncinate process of pancreas (middle). Note that the photo has been taken intraoperatively

**Fig. 3.**
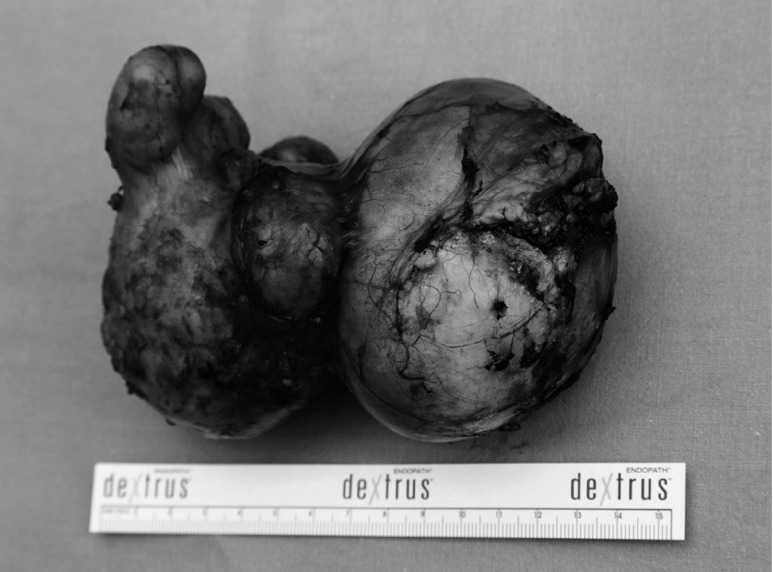
Image of the removed specimen

During the exploration, a cystic mass with different sizes of daughter cysts was found in the uncinate process of pancreas infiltrating the retroperitoneum *(Fig. 2)*. The cystic lesion was enucleated and the uncinate process of pancreas was resecated *(Fig. 3)*. After an eventless postoperative period, patient was discharged from hospital on the 7^th^ day after the surgical procedure.

Pathological examination of the enucleated lesion confirmed the diagnosis of hydatid disease. Patient received albendazole treatment during follow-up. He had no symptoms and complaints 6 months after the operation.

## Discussion

In hydatid disease, liver is involved in 60%–70% of all cases, followed by the lungs (10%–25%) and in sporadic cases in other organs [1]. Primary pancreas hydatid disease is extremely rare, with an incidence of 0.2%–2% of all cases [1–3, 5]. Hydatid cysts are soliter in 90% of all cases [6]. The authors enucleated multivesicular hydatid cysts.

The patient in our case was also almost asymptomatic. Most frequent complaint is epigastric pain or feeling of repletion. Localization in the pancreas head or large cysts can cause jaundice, portal hypertension, and rarely acute pancreatitis, and rupture of the cysts can cause anaphylactic shock [6, 7].

In the presented case, the serological test was negative that is not unique in the literature [3]. In typical case, hydatid cysts are multivesicular, have a sharp wind and wall calcification, and daughter cysts may also be observable by radiological images [1]. Differential diagnosis of hydatid disease is complicated, because hydatid cysts can be confused with more other frequent cystic diseases of pancreas [8]. Therefore, if the diagnosis is not secured, a surgical procedure is suggested.

Surgical procedures are primary in the treatment of pancreatic hydatid disease. Hydatid cysts in the pancreas corpus or tail can be treated by pericystectomy, central pancreatectomy, and distal pancreatectomy with or without splenectomy [3, 7]. When cyst is located in the head region, evacuation of cyst fluid, partial cystectomy, marsupialization, or surgical drainage can be chosen [2, 3]. Partial pancreatectomy is necessary when the possibility of neoplastic cysts cannot be excluded [1]. In the presented case, the cyst was removed by the partial resection of uncinate process of pancreas. Concerning that cysts located in the head of pancreas frequently cause jaundice, many authors recommend T-tube drainage [3]. Diagnosis can be verified by radiological examinations (US or CT scan), percutaneous puncture of the cyst, and then injection of contrast mediums [9]. Bedioui et al. [10] recommend intraoperative cholangiopancreatography to identify the communication between the cyst and the pancreatic duct.

Resection is also recommended when one cannot exclude securely the possibility of neoplasia. An alternative treatment can be the percutan cyst puncture, aspiration, injection of hypertonic saline solution or absolute alcohol, then reaspiration, or placing a pig-tail catheter into the cyst [9]. Treatment should be completed with albendazole therapy, which is already recommended preoperatively [9].

When pancreatic fistula develops after the surgical procedure, the fistula can be sutured, but cystogastrostomy or Rouen-Y pancreaticojejunostomy can also be made [11]. Because of the increased possibility of pancreatic fistulas after pericystectomy, some authors recommend resection in order to prevent the development of fistulas [7, 10].

A review that analyzed eight cases of hydatid disease causing pancreatitis does not report recurrence in any patient [7]. Another publication that elaborated different surgical techniques in six cases also does not report any recurrences, but the authors recommended resection in order to minimize the risk of recurrence if patient’s condition made it feasible. [3].

In conclusion, primary pancreatic hydatid disease is rare. Caution is needed when cysts in the pancreas are identified in good conditioned patients, the disease is asymptomatic, or in case of wall calcification or multivesicular cysts revealed by radiological images. Surgical procedures are recommended in uncertain diagnoses, because differentiation between hydatid cysts, pseudocysts, and neoplastic cysts can often be confirmed only by pathological examination of the resecated cyst wall.
